# Advancing Interprofessional Palliative Care Education: From Needs Assessment to Pilot Testing

**DOI:** 10.1093/geroni/igaf122.3369

**Published:** 2025-12-31

**Authors:** Innessa Manning, Christina Matz, Anna Kennedy, Patricia Tabloski, Karen Bullock

**Affiliations:** Boston College, Lexington, Massachusetts, United States; Boston College, Chestnut Hill, Massachusetts, United States; Dana-Farber Cancer Institute, Boston, Massachusetts, United States; Boston College, Chestnut Hill, Massachusetts, United States; Boston College, Chestnut Hill, Massachusetts, United States

## Abstract

Interprofessional education is essential in preparing health professionals for effective team-based practice, particularly in palliative care, where interdisciplinary collaboration is fundamental. This poster presents findings from a qualitative needs assessment and pilot testing of a newly developed graduate-level palliative care curriculum to enhance interdisciplinary training for social work, nursing, and theology students. A needs assessment survey (n = 25) conducted with palliative care professionals identified key competencies necessary for effective interprofessional practice, including communication, role clarity, and cultural competence. Structural and systemic challenges such as discipline-specific silos, inconsistent training, and workforce development gaps were also highlighted, emphasizing the need for targeted curriculum interventions that promote team-based care and cultural responsiveness. In response, an interdisciplinary faculty team developed a nine-module asynchronous online course designed to: (1) build discipline-specific expertise within a team context, (2) foster cross-disciplinary collaboration, (3) integrate culturally responsive approaches to patient and family engagement, and (4) promote sustainable practices to mitigate clinician burnout. Pilot testing with a Student Advisory Group—comprising graduate students from social work, nursing, and theology—provided feedback on content effectiveness, interdisciplinary integration, and applicability to real-world palliative care settings. Findings from the needs assessment and pilot testing underscore the importance of interdisciplinary education in developing a workforce capable of delivering compassionate, holistic palliative care. This initiative offers a replicable model for interprofessional training, equipping future clinicians with the knowledge and skills necessary to meet the growing demands of an aging and diverse patient population. Focus areas: interprofessional education, palliative care, interdisciplinary collaboration, curriculum development, cultural responsiveness, workforce training

